# Comparative transcriptome analysis revealed key factors for differential cadmium transport and retention in roots of two contrasting peanut cultivars

**DOI:** 10.1186/s12864-018-5304-7

**Published:** 2018-12-17

**Authors:** Rugang Yu, Yuanyuan Ma, Yue Li, Xin Li, Caifeng Liu, Xueling Du, Gangrong Shi

**Affiliations:** College of Life Sciences, Huaibei Normal University, Huaibei, Anhui 235000 People’s Republic of China

**Keywords:** Peanut, Root, Cadmium, Transcriptome, Gene expression

## Abstract

**Background:**

Peanut is the world’s fourth largest oilseed crop that exhibits wide cultivar variations in cadmium (Cd) accumulation. To establish the mechanisms of Cd distribution and accumulation in peanut plants, eight cDNA libraries from the roots of two contrasting cultivars, Fenghua 1 (low-Cd cultivar) and Silihong (high-Cd cultivar), were constructed and sequenced by RNA-sequencing. The expression patterns of 16 candidate DEGs were validated by RT-qPCR analysis.

**Results:**

A total of 75,634 genes including 71,349 known genes and 4484 novel genes were identified in eight cDNA libraries, among which 6798 genes were found to be Cd-responsive DEGs and/or DEGs between these two cultivars. Interestingly, 183 DEGs encoding ion transport related proteins and 260 DEGs encoding cell wall related proteins were identified. Among these DEGs, nine metal transporter genes (*PDR1*, *ABCC4* and *ABCC15*, *IRT1*, *ZIP1*, *ZIP11*, *YSL7*, *DTX43* and *MTP4*) and nine cell wall related genes (*PEs*, *PGIPs*, *GTs*, *XYT12 CYP450s*, *LACs*, *4CL2*, *C4H* and *CASP5*) showed higher expression in Fenghua 1 than in Silihong.

**Conclusions:**

Both the metal transporters and cell wall modification might be responsible for the difference in Cd accumulation and translocation between Fenghua 1 and Silihong. These findings would be useful for further functional analysis, and reveal the molecular mechanism responsible for genotype difference in Cd accumulation.

**Electronic supplementary material:**

The online version of this article (10.1186/s12864-018-5304-7) contains supplementary material, which is available to authorized users.

## Background

Cadmium (Cd) is one of the most widespread and toxic heavy metal pollutants, whose contamination in arable soil from natural and anthropogenic sources has drawn wide public health concern [1]. Although Cd is a non-essential element for plant growth, it is easily taken up by the root system and transferred to aboveground parts of plants [2]. The excess amount of Cd in plants can trigger negative effects on many physiological processes such as inhibition of photosynthesis, interference of nutrient uptake and induction of oxidative stress [3]. Moreover, Cd can accumulate in the human body via the food chain, posing various health problems including *itai*-*itai*, cancer, renal dysfunction, osteoporosis and cardiovascular diseases [2]. It is very important to take effective measures to reduce Cd accumulation in edible parts of crops. For this purpose, screening and breeding low-Cd cultivars are expected to be one of the sustainable strategies [4].

Peanut (*Arachis hypogaea* L.; AABB, 2n = 4x = 40) is an important oilseed crop that is cultivated extensively worldwide. China is one of the major exporters of peanut products, sharing around 40% of the world trade in peanut products [5]. However, higher Cd enrichment capacity in the seeds of peanuts has become a serious problem for the peanut industry [6–9]. Seed Cd contents of peanuts in some regions of China such as Shandong and Liaoning have been shown to exceed the maximum level of the contaminant (0.5 mg/kg for Cd) following Chinese standard (GB 2762–2012) [10]. Moreover, the higher Cd accumulation in peanut seeds has also been reported in other countries, such as Australia [9, 11] and Argentina [12]. Exposure of plants to 2 and 4 mg kg^− 1^ Cd did not inhibit shoot biomass, seed yield, and oil content for most of the peanut cultivars [13]. Thus, cultivation of peanuts in Cd contaminated soil may pose severe health risks for human beings.

Peanuts exhibit a wide variation in Cd accumulation in vegetative organs and seeds among cultivars [6–8, 13, 14]. Seed Cd concentrations of peanuts are not associated with Cd concentrations in vegetative organs at the mature stage, but positively correlated with Cd concentrations in shoots of the seedlings [6, 8]. The results suggest that shoot Cd concentrations at the vegetative growth stage may be important for determining cultivar differences in Cd accumulation in the seed [6, 8]. Although peanut seeds develop in the soil, their Cd concentration predominantly comes from the uptake of Cd by the root system [7, 9, 15]. Cd immobilization in the peanut root system could inhibit Cd translocation in peanut plants, and reduce Cd accumulation in peanut seed [16, 17]. Root morphology has been shown to significantly correlate with Cd accumulation in peanuts [14]. Cultivars with longer fine roots have a high capacity of Cd accumulation [14]. It seems that the root plays an important role in determining variation in Cd accumulation among peanut cultivars, however, the underlying mechanism is still unclear. Understanding the molecular mechanism of cultivar difference in Cd accumulation is essential for breeding low Cd cultivars of peanuts.

The uptake and translocation of Cd in plant is controlled by some metal transporter proteins such as natural resistance associated macrophage proteins (Nramp) [18, 19], zinc-regulated transporters, iron-regulated transporter-like protein (ZIP) [20], ATP-binding cassette transporters (ABC) [21], and P_1B_-ATPases [22, 23]. Furthermore, the translocation of Cd from roots to shoots was also determined by the capacity for retaining Cd in the roots, in which Cd binding to the cell wall and vacuolar Cd sequestration are involved [24, 25]. Therefore, we hypothesize that cultivar differences in Cd accumulation in peanut may be resulted from the differential expression of genes encoding metal transporters and cell wall modification in the roots.

In this study, a comparative transcriptome analysis was carried out on the roots of two peanut cultivars differing in seed Cd accumulation, Silihong (high Cd-accumulating cultivar) and Fenghua 1 (low Cd-accumulating cultivar) [8], under Cd-free and Cd-treated conditions. The main aims are: (i) to reveal the gene expression patterns in the roots of Fenghua 1 and Silihong in response to Cd exposure; (ii) to identify the key genes involved in Cd uptake, distribution and translocation in peanut roots; and (iii) to elucidate the gene regulatory network that is responsible for the cultivar differences of peanuts. The results presented here would be important for further illustrating the mechanism of Cd uptake and translocation as well as for breeding low Cd cultivars of peanuts.

## Results

### Cd accumulation and translocation in plants

**Fig. 1:**
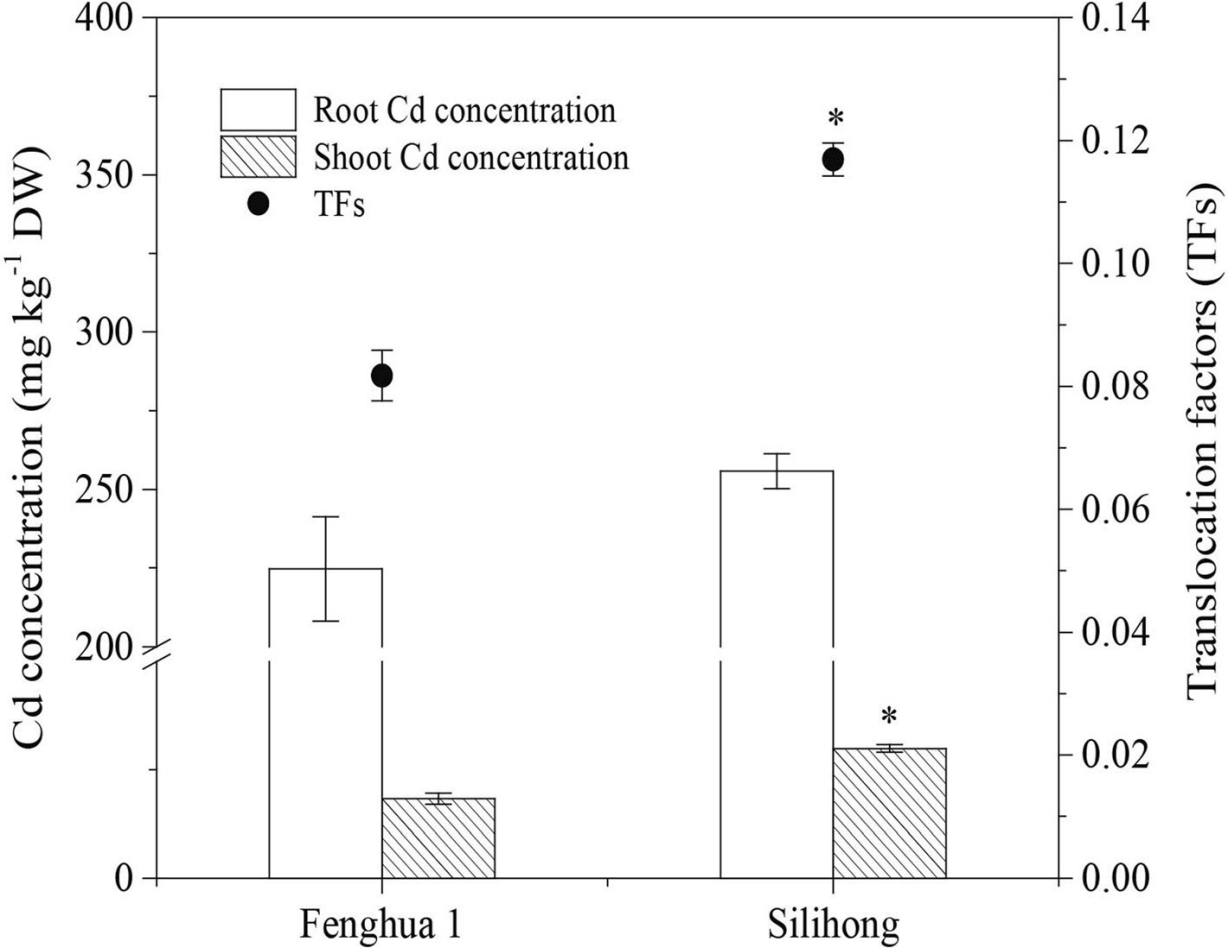
Cd accumulation and translocation in plants of Fenghua 1 and Silihong exposed to 2 μM Cd for seven days. Asterisk (*) above error bars indicate values (mean ± SE, *n* = 4) are significantly different between the two peanut cultivars according to independent samples t-test at 0.05 the level

When exposed to 2 μM Cd for seven days, the concentration of Cd in roots and shoots was 224.7 and 18.3 mg kg^− 1^ dry weight for Fenghua 1, and 255.8 and 29.9 mg kg^− 1^ dry weight for Silihong, respectively (Fig. 1). Shoot Cd concentration was significantly higher in Silihong than in Fenghua 1, while the root Cd concentration was similar between the two cultivars. Similar to the shoot Cd concentration, the translocation factor of Cd from roots to shoots was significantly high in Silihong (0.12) than in Fenghua 1 (0.08) (Fig. 1). The current results, in agreement with previous findings [8], indicate that Fenghua 1 differed in the properties of Cd translocation and accumulation from Silihong.

### RNA sequencing analysis of eight cDNA libraries

Eight cDNA libraries, namely, F_CK__1, F_CK__2, F_Cd__1, F_Cd__2, S_CK__1, S_CK__2, S_Cd__1 and S_Cd__2, were constructed from Cd-free (CK) and Cd-treated (Cd) peanut roots of both cultivars and sequenced using a BGISEQ-500 platform. A total of 164.87, 167.18, 164.59 and 167.03 Mb raw reads were produced from two biological replicate libraries for F_CK_, F_Cd_, S_CK_ and S_Cd_, respectively. After removing the reads with adapters, low-quality and high content of unknown base (N), more than 63 million 100 bp paired-end clean reads with Q20 percentage over 97% remained in each cDNA library (Additional file 1: Table S1).

The clean reads from each cDNA library were aligned to reference genome sequences of the two progenitors of the cultivated peanut (*A. duranensis*-AA and *A. ipaensis*-BB). The average genome mapping rate is 90.16%, and the average gene mapping rate is 86.05% (Additional file 2: Table S2).

### Analysis of gene expression levels

A total of 75,634 genes including 71,349 known genes and 4484 novel genes were identified from A and B genome (Additional file 3: Table S3 and Additional file 4: Table S4). Furthermore, 24,024 novel isoforms and 5264 noncoding transcripts were identified in eight cDNA libraries (Additional file 3: Table S3). The expression levels of these genes from the mapped libraries were normalized as FPKM (Additional file 5: Figure S1). The gene expression distribution (FPKM) was similar among eight libraries. For instance, the low expression level genes with FPKM interval 0~10 account for 63.50, 64.62, 64.66, 63.41, 64.04, 63.36, 59.24 and 63.56% in F_Cd__1, F_Cd__2, F_CK__1, F_CK__2, S_Cd__1, S_Cd__2, S_CK__1 and S_CK__2 respectively.

### Identification of differentially expressed genes (DEGs)

**Fig. 2:**
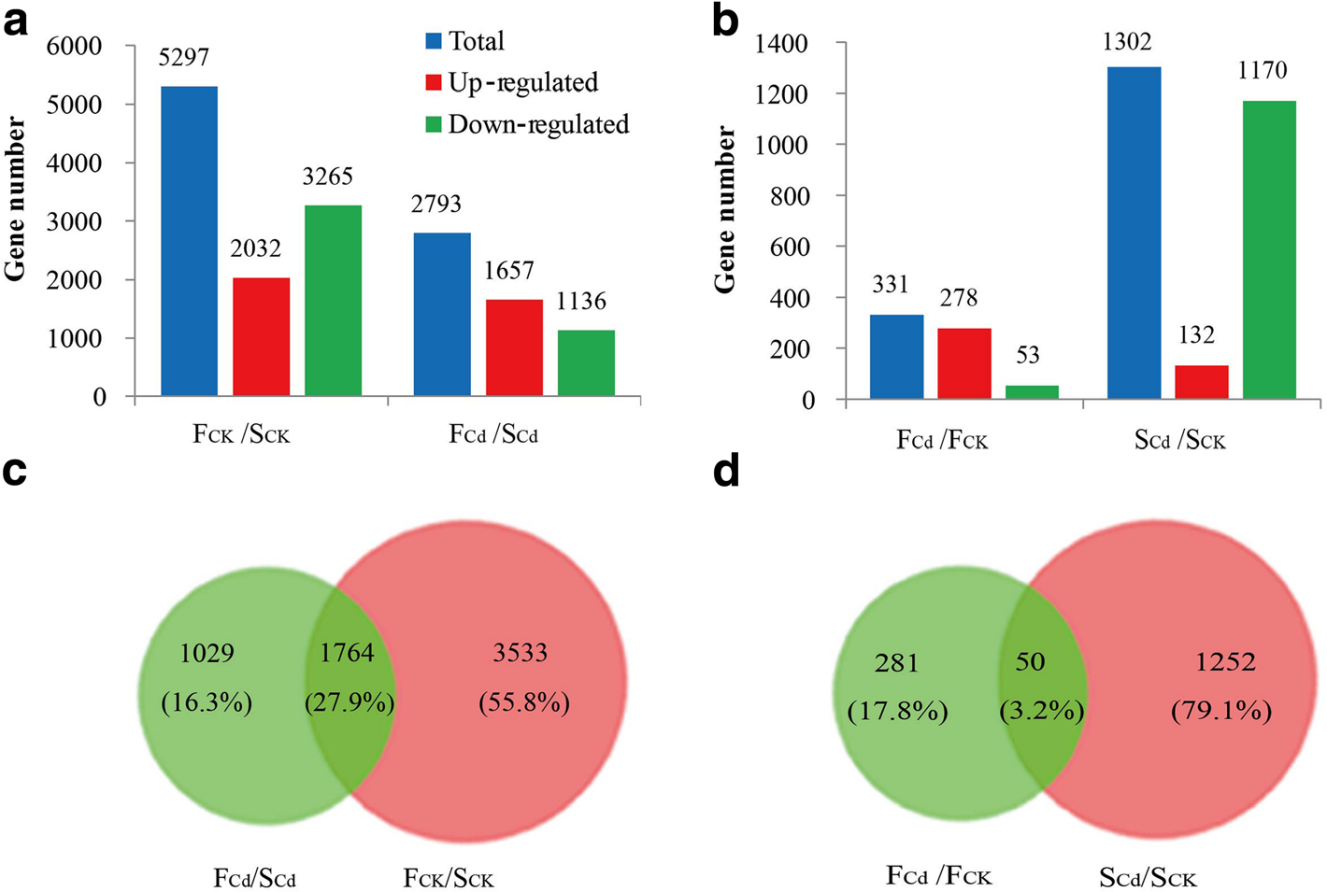
Analysis of DEGs following Cd exposure in two peanut cultivars. (**a**) Number of DEGs between Fenghua 1 (F) and Silihong (S). (**b**) Number of DEGs between the control (CK) and Cd exposure (Cd). (**c**) Venn diagrams of DEGs between Fenghua 1 (F) and Silihong (S). (**d**) Venn diagrams of DEGs between the control (CK) and Cd exposure (Cd)

Pairwise comparison analysis for each gene were performed between Fenghua 1 and Silihong (F_CK_/S_CK_ and F_Cd_/S_Cd_) or between Cd-free and Cd-treated samples in each cultivar (F_Cd_/F_CK_ and S_Cd_/S_CK_). DEGs were identified by the threshold of |log_2_ fold-change| ≥ 1 and *P*_adj_-value ≤ 0.05. A total of 6798 genes were differentially regulated in the four comparisons (Additional file 6: Table S5). Under control condition, 5297 DEGs were identified between two cultivars, while this value reached 2793 DEGs under Cd-treated condition (Fig. 2a). Of which, 1764 genes were common ones (Fig. 2c). Compared to Cd-free library, 331 genes were differentially regulated in Fenghua 1 in Cd exposure, including 278 up-regulated and 53 down-regulated genes. Whereas, 1302 Cd-treated DEGs were identified in Silihong, including 132 up-regulated and 1170 down-regulated genes (Fig. 2b). Among these genes, only 50 DEGs were common Cd-responsive genes in two cultivars (Fig. 2d), including 49 genes showed similar expression patterns and one gene showed opposing expression pattern (Additional file 6: Table S5).

### Gene ontology (GO) analysis of DEGs

GO assignments were used to classify the functions of DEGs, a total of 2860 genes including 157 (F_Cd_/F_CK_), 610 (S_Cd_/S_CK_), 2214 (F_CK_/S_CK_) and 941 (F_Cd_/S_Cd_) genes, were assigned into 46 GO terms consisting of 22 biological process, 13 cellular component and 11 molecular function subcategories (Additional file 7: Figure S2a-b). Among these GO terms, the top two abundant categories of biological process (‘metabolic process’ and ‘cellular process’), molecular function (‘catalytic activity’ and ‘binding’), and cellular component (‘membrane’ and ‘membrane part’) were similar in the four comparisons.

Furthermore, the results of significantly enriched GO terms (corrected *p*-value < 0.01) are listed in Additional file 8: Table S6. For Cd-responsive DEGs, four GO terms such as response to inorganic substance, response to symbiont and response to metal ion in the biological process category and 16 GO terms such as zinc ion transmembrane transporter activity, divalent inorganic cation transmembrane transporter activity and transporter activity in the molecular function category were uniquely enriched in Fenghua 1. In Silihong, 11 GO terms such as transcription, DNA-templated, RNA biosynthetic process and nucleic acid-templated transcription in the biological process category and ten GO terms such as DNA binding transcription factor activity, transcription regulator activity and calcium ion binding in the molecular function category were uniquely enriched (Additional file 8: Table S6). For DEGs between Fenghua 1 and Silihong, three GO terms including lignin metabolic process, phenylpropanoid metabolic process and lignin catabolic process in the biological process category and the two GO terms including hydroquinone: oxygen oxidoreductase activity and oxidoreductase activity, acting on diphenols and related substances as donors, oxygen as acceptor (Additional file 8: Table S6) were significantly enriched under Cd-treated condition.

### KEEG metabolic pathway analysis of DEGs

**Fig. 3:**
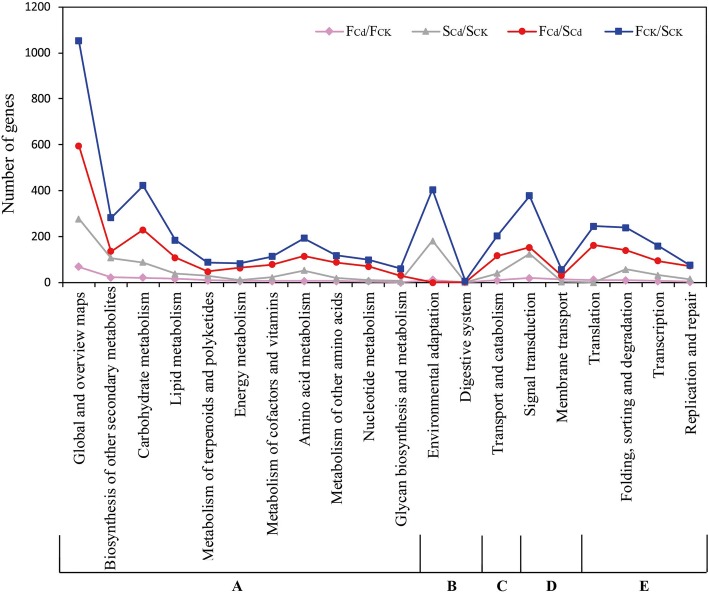
Functional classification and pathway assignment of identified DEGs by KEEG in four comparisons. The DEGs were assigned pathways belonging to five main categories: Metabolism (**a**), Organismal systems (**b**), Cellular processes (**c**), Environmental information processing (**d**), and Genetic information processing (**e**)

Here, DEGs were further annotated with Kyoto Encyclopedia of Genes and Genomes (KEGG) database (http://www.genome.ad.jp/kegg/) to elucidate the molecular interactions among the genes. A total of 266 (F_Cd_/F_CK_), 1073 (S_Cd_/S_CK_), 4164 (F_CK_/S_CK_) and 2115 (F_Cd_/S_Cd_) DEGs were respectively assigned to 66, 116, 133, and 132 pathways (Additional file 9: Table S7), which were grouped into five groups including metabolism, genetic information processing, environmental information processing, cellular processes and organismal systems (Fig. 3). Among these pathways, the top two categories of them including metabolic pathway (59, 22.18%; 254, 23.67%; 960, 23.05%; 535, 25.30%) and biosynthesis of secondary metabolites (46, 17.29%; 193, 17.99%; 617, 14.82%; 337, 15.93%) were similar in the four comparisons (Additional file 9: Table S7). Followed by MAPK signaling pathway-plant, phenylpropanoid biosynthesis and ABC transporters in F_Cd_/F_CK_; plant-pathogen interaction, MAPK signaling pathway-plant and flavonoid biosynthesis in S_Cd_/S_CK_; Plant-pathogen interaction, MAPK signaling pathway-plant and plant hormone signal transduction in F_CK_/S_CK_; plant-pathogen interaction, RNA transport, protein processing in endoplasmic reticulum and phenylpropanoid biosynthesis in F_Cd_/S_Cd_ (Additional file 9: Table S7).

In addition, one (F_Cd_/F_CK_), six (S_Cd_/S_CK_), four (F_CK_/S_CK_) and two (F_Cd_/S_Cd_) pathways were identified as significantly enriched (Q ≤ 0.01) (Additional file 10: Table S8). For DEGs between Fenghua 1 and Silihong, circadian rhythm-plant pathway was shared enriched under both control and Cd conditions (Additional file 10: Table S8). Besides, the pathway of cyanoamino acid metabolism was specifically significantly enriched for DEGs between two cultivars under Cd-treated. Further, Cd-responsive DEGs in Fenghua 1 were specifically significantly enriched in ABC transporters. The pathways of alpha-Linolenic acid metabolism, plant-pathogen interaction, flavonoid biosynthesis, circadian rhythm-plant, zeatin biosynthesis and MAPK signaling pathway-plant were significantly enriched for Cd-responsive DEGs in Silihong.

### High expression level of DEGs between Fenghua 1 and Silihong under Cd exposure

To obtain a general statistical overview of transcriptional gene regulation the difference of shoot Cd accumulation in two peanut cultivars, the high expression level of DEGs (an FPKM value > 10 in at least one of four cDNA libraries and the threshold of |log_2_ (F_Cd_/S_Cd_)| ≥ 3) including 109 up- and 96 down-regulated were identified in F_Cd_/S_Cd_ (Additional file 11: Table S9). Based on functional analysis, some of them were involved in cell wall metabolism (i.e., polygalacturonase inhibitory PGI, chitinase-like protein and GDSL esterase/lipase), ion transport (i.e., ABC transporter D family member 1 ABCD1, V-type proton ATPase), transcription regualtion (i.e., transcription factor bHLH83, bZIP transcription factor 11 and auxin-induced protein AUX28) and other metabolic processes (i.e., serine/threonine-protein kinase, metallothionein-like protein). The results suggested that these DEGs might play crucial regulatory roles in the the difference of shoot Cd accumulation in two peanut cultivars. Here, the DEGs were involved in the cell wall metabolism and ion transport as our main further investigation.

### DEGs involved in heavy metal transport

According to GO functional annotation, 183 DEGs were identified to have high similarity with diverse transporters such as ATP-binding cassette (ABC) transporters (e.g. ABCAs, ABCBs, ABCCs and ABCGs), natural resistance-associated macrophage proteins (e.g. Nramp5 and Nramp6), ZIP transporters (e.g. IRT1, IRT3, ZIP1, ZIP4, ZIP5 and ZIP11), yellow stripe-like transporters (e.g. YSL3 and YSL7), MATE efflux family proteins (e.g. protein DETOXIFICATIONs DTXs) and metal tolerance proteins (e.g. MTP4 and MTP9) (Additional file 12: Table S10). These transporters were classified into seven categories based on their expression patterns (Additional file 13: Table S11). The classes from first to fourth were Cd-responsive DEGs. Of these, two genes in the first class were up-regulated, and one gene in the second class was down-regulated in both Fenghua 1 and Silihong after Cd exposure; the 22 genes in the third class and five genes in the fourth class were up-regulated in Fenghua 1 and Silihong roots under Cd exposure, respectively. Furthermore, the fifth and sixth classes were DEGs between two cultivars. The fifth class contained 58 genes whose expressions were higher in Fenghua 1 than those in Silihong, whereas, the sixth class had 61 genes expressed lower in Fenghua 1 than those in Silihong. The 34 genes in seventh class were Cd-responsive DEGs that is also differentially expressed between Fenghua 1 and Silihong.

We also found that the DEGs encoding heavy metal-mediated transporters, such as ABCs, IRTs, ZIPs, YSL3, Nramp5, MTP9, pleiotropic drug resistance proteins (PDRs) and copper-transporting ATPase HMA5 (HMA5), were all up-regulated by Cd in Fenghua 1 (Additional file 12: Table S10). In S_Cd_/S_CK_, besides the DEGs encoding transporters including ABCB15, ABCG20, HMA5, Nramp6, DTX40 and two pore calcium channel protein 1 (TPCN1), the remaining 22 DEGs encoding transporters such as heavy metal-associated isoprenylated plant proteins (HIPPs), calcium-transporting ATPase 2 (Ca^2+^-ATPase2), MATE efflux family protein (MATE), and ABCG11, were down-regulated after Cd exposure (Additional file 12: Table S10). For the F_Cd_/S_Cd_, genes encoding ABC transporters (e.g. ABCB28, ABCC4/8/15, ABCD1 and ABCG15), oligopeptide transporter 7 (OPT7), DTX43/49, IRT1, PDR1, YSL7 and MTP4, were higher in Fenghua 1 than in Silihong. However, ABCA1, ABCB1/11, ABCC10, ABCG7, HIPP35, ZIP1, Nramp2, Ca^2+^-ATPase12, cation/H(+) antiporter (CHX18/20), DTX16/43/48/51 and MATE expressed higher in Silihong than in Fenghua 1 (Additional file 12: Table S10).

### DEGs related to cell wall modification and vacuolar sequestration

Plant cell wall has been considered as the critical site for retention of Cd, especially in roots. Our study suggested that a total of 260 DEGs were homologous with cell wall metabolism-related genes, including 108 involved in cell wall degradation (75, 23 and five were involved in pectin-, hemicellulose- and cellulose-degrading process, respectively; five were other cell wall degrading-related enzymes), 143 involved in cell wall synthesis (53 in cellulose biosynthesis, 49 in suberin biosynthesis, 24 in lignin biosynthesis, 11 in hemicellulose, four in pectin, and two in Casparian strip formation). Furthermore, three encoding expansin proteins, five encoding COBRA proteins and one encoding extension 2 like protein were involved in cell wall organization (Additional file 14: Table S12).

For the 108 cell wall degradation DEGs, six were Cd-responsive DEGs including one upregulated in Fenghua 1 and five downregulated in Silihong, and 87 were DEGs between Fenghua 1 and Silihong (Additional file 14: Table S12). The remaining 15 genes were Cd responsive DEGs, which also expressed differentially between two cultivars, and 13 of them were down-regulated by Cd in Silihong (Additional file 14: Table S12). Furthermore, among 108 DEGs, only three upregulated were found in F_Cd_/F_CK_. Remarkably, 32 of 108 DEGs were expressed higher in Fenghua 1 than in Silihong. Regarding the 143 cell wall synthesis DEGs, 19 were Cd-responsive DEGs and 97 were DEGs between Fenghua 1 and Silihong. For the 19 Cd responsive DEGs, 15 were up-regulated by Cd in Fenghua 1, and four were down-regulated by Cd in Silihong. The remaining 27 genes were Cd responsive DEGs, which also expressed differentially between two cultivars. 46 of 143 DEGs were expressed higher in Fenghua 1.

In addition, the formation of Cd complexes with glutathione (GSH), metallothionein (MT), phytochelatin (PC) and nicotianamine (NA) is another way used by plants to sequestrate Cd in vacuolar. In this study, two (107459596, 107612042) and one (107465178) nicotianamine synthase (NAS) genes were up-regulated by Cd in Fenghua 1 and Silihong, respectively. Moreover, NAS (107612042) and MT2 (107639467) were expressed higher in Fenghua 1 than in Silihong (Additional file 14: Table S12). However, no significant difference in GSH and PC biosynthesis-related genes expression were observed in each comparison.

### RT-qPCR validation

**Fig. 4:**
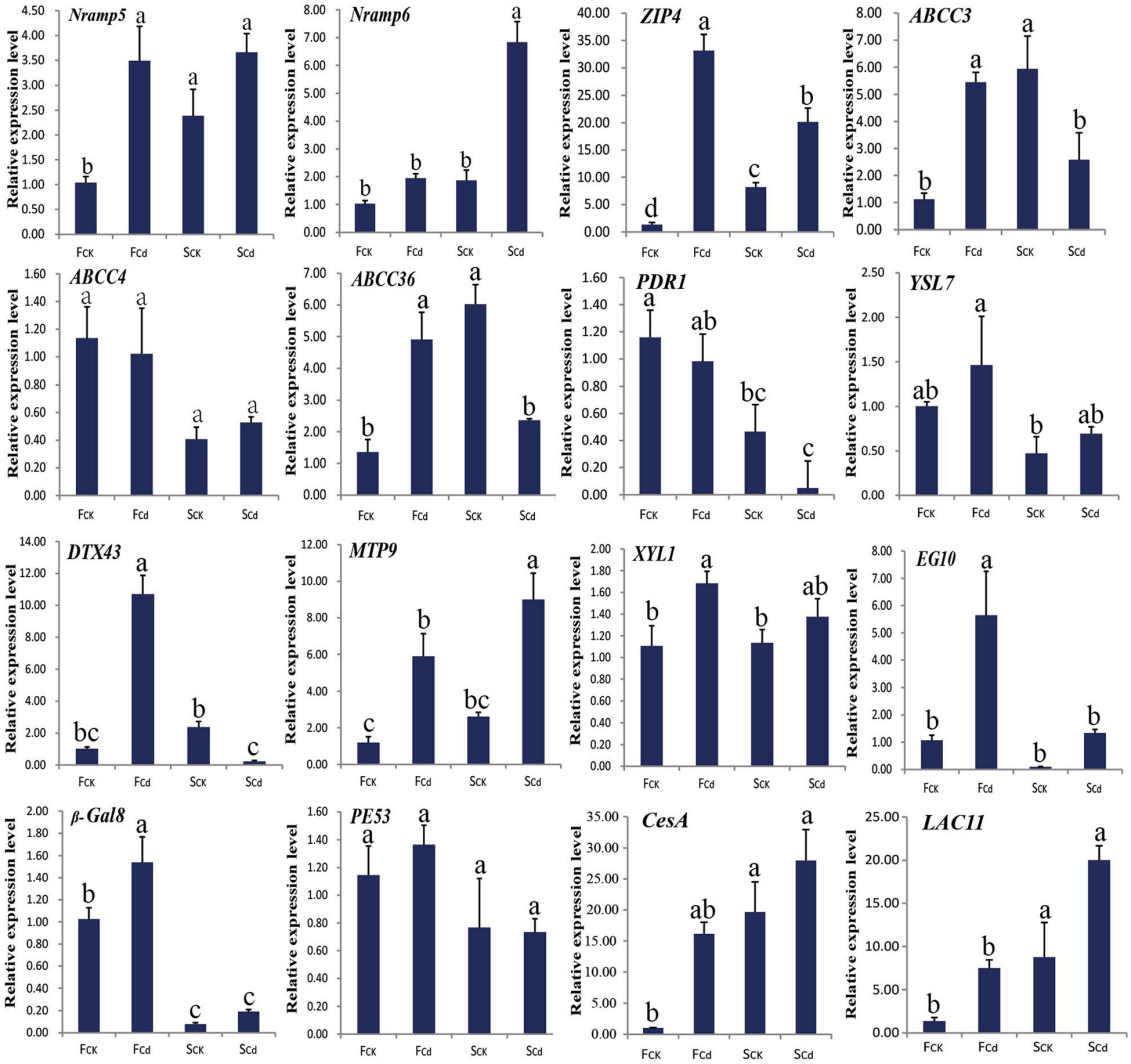
RT-qPCR analyses of 16 candidate DEGs under the control and Cd treatment in roots of two peanut cultivars. Each bar represents the mean ± SD of triplicate assays. The values with different letters indicate significant differences at *P* < 0.05 according to Duncan’s multiple range tests

To verify the differential expression patterns of DEGs identified in our transcriptome data, a total of ten candidate transport- and six cell wall-related genes were selected for RT-qPCR analysis. The results indicated that the expression patterns of 15 out of 16 DEGs showed good agreement with the RNA-Seq-based gene expression patterns, and only one gene (*ABCC36*) was not well consistent with the results of sequencing (Fig. 4; Additional file 6: Table S5). For example, several Cd-responsive up-regulated DEGs also indicated relatively high expression level in Fenghua 1 (*ZIP4*, *Nramp5/6*, and *ABCC3*) and Silihong (*Nramp6*) under Cd exposure in RT-qPCR analysis (Fig. 4). For DEGs between Fenghua 1 and Silihong, the expression level of *ABCC4*, *PDR1*, *DTX43*, *β-Gal8*, *XYL1*, *EG10* genes were higher in Fenghua 1, whereas *LAC11* was highly expressed in Silihong under Cd exposure (Fig. 4). These results suggested that the data detected by RNA-Seq analysis of this study provide reliable inference.

## Discussion

### Comparison of gene expression in roots between Fenghua 1 and Silihong

There are clear physiological differences in shoot Cd concentrations between Fenghua 1 and Silihong following Cd exposure [8]. However, the molecular mechanism underlying the difference in Cd accumulation among peanut cultivars has not been fully elucidated.

RNA sequencing (RNA-seq) analysis has been used for revealing the molecular mechanisms of differential Cd uptake, accumulation and translocation in many plants [26–28]. However, the comprehensive identification of the DEGs involved in root uptake, accumulation and transport of Cd in peanut remained unexplored. Based on peanut genome [29], a total of 6798 genes including 331 (F_CK_/F_Cd_), 1302 (S_CK_/S_Cd_), 5297 (F_CK_/S_CK_) and 2793 (F_Cd_/S_Cd_) genes, were identified significant differentially expression in pairwise comparisons (Additional file 6: Table S5). Of them, 183 DEGs encoding transporter proteins and 260 DEGs encoding cell wall metabolism-related proteins were identified. Most Cd-responsive DEGs (278/331, 83.99%) in Fenghua 1 were up-regulated by Cd, while the majority of Cd-responsive DEGs (1170/1302, 89.86%) in Silihong were down-regulated by Cd (Additional file 6: Table S5). The results suggested that the two cultivars differed in the molecular mechanisms in response to Cd exposure. To our knowledge, this is the first research to identify and dissect extensive Cd-responsive genes in peanuts, and insight into molecular mechanisms responsible for the differential shoot Cd accumulation in two cultivars.

### Heavy metal transporters involved in differential root uptake and translocation of Cd between Fenghua 1 and Silihong

It has been widely proven that Zn^2+^, Ca^2+^, Fe^2+^, Mn^2+^, Cu^2+^ and Mg^2+^ transporters could transport Cd into plant root cells, and affect root-to-shoot Cd translocation [30]. The physiological data indicated that Fenghua 1 showed lower shoot Cd concentrations than Silihong, for which the root-to-shoot translocation of Cd might be responsible [8]. In this study, we identified 183 DEGs encoding ion transporter proteins, among which 30 were Cd-responsive DEGs. We also found that 34 Cd-responsive DEGs encoding ion transporter proteins were expressed differentially between the two cultivars (Additional file 12: Table S10). Compared with the control, Cd up-regulated the Cd-responsive DEGs in Fenghua 1, whereas most of DEGs in Silihong were down-regulated by Cd. Only four DEGs, 107471962 and 107623484 (HMA5), 107477738 (DTX40) and 107491040 (HIPP2), were induced by Cd treatment in both cultivars. These results indicate that the two peanut cultivars differed in the expression of ion transporter that may contribute to the difference in Cd uptake and translocation.

**Fig. 5:**
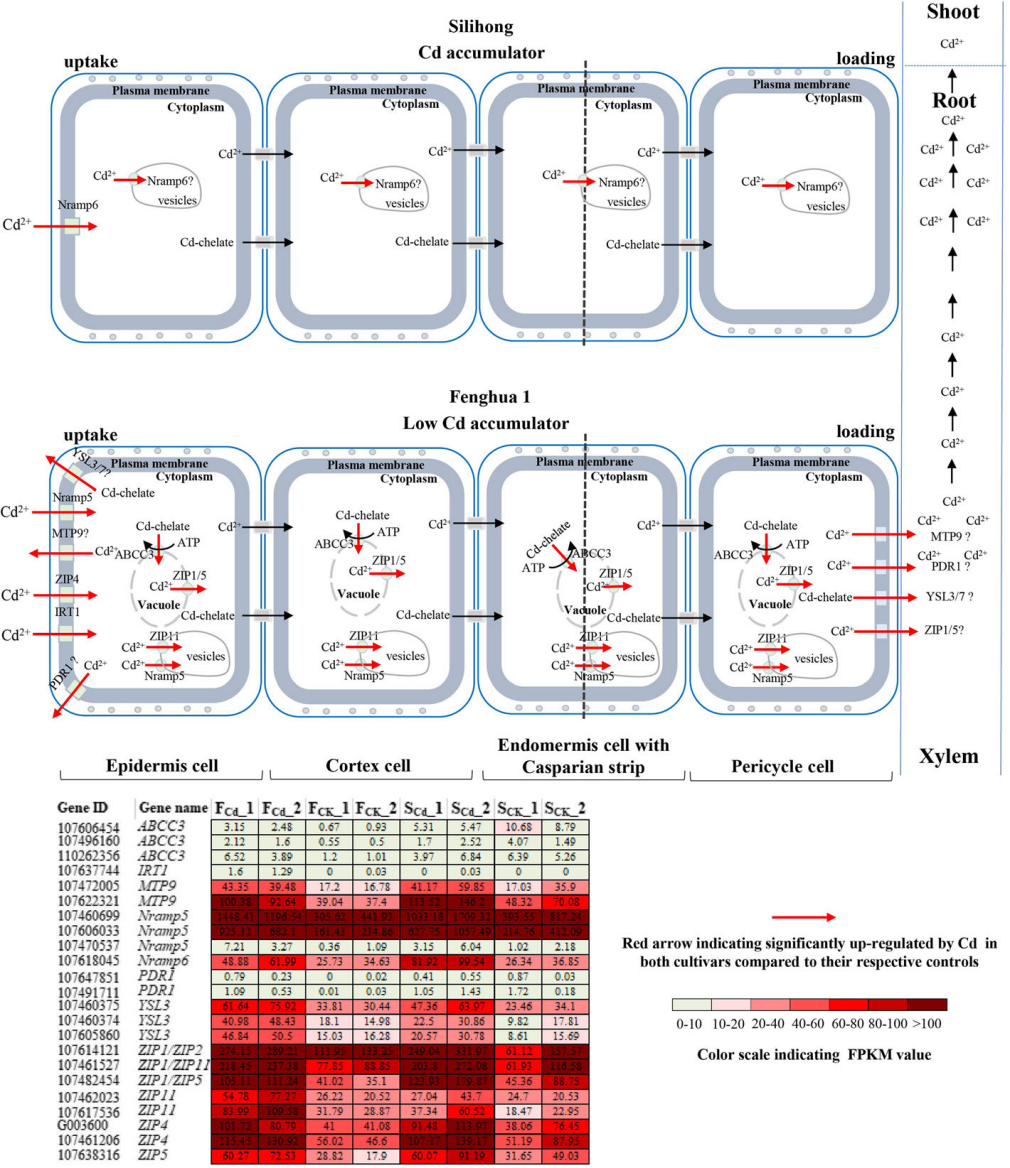
The putative model of regulatory networks associated with transport genes in the regulation of Cd translocation and compartmentation in roots of two peanut cultivars. The grid representing the FPKM value of each gene in eight samples, respectively. The localization of these genes was based on the data of Arabidopsis, rice and other species (see Table 1)

**Fig. 6:**
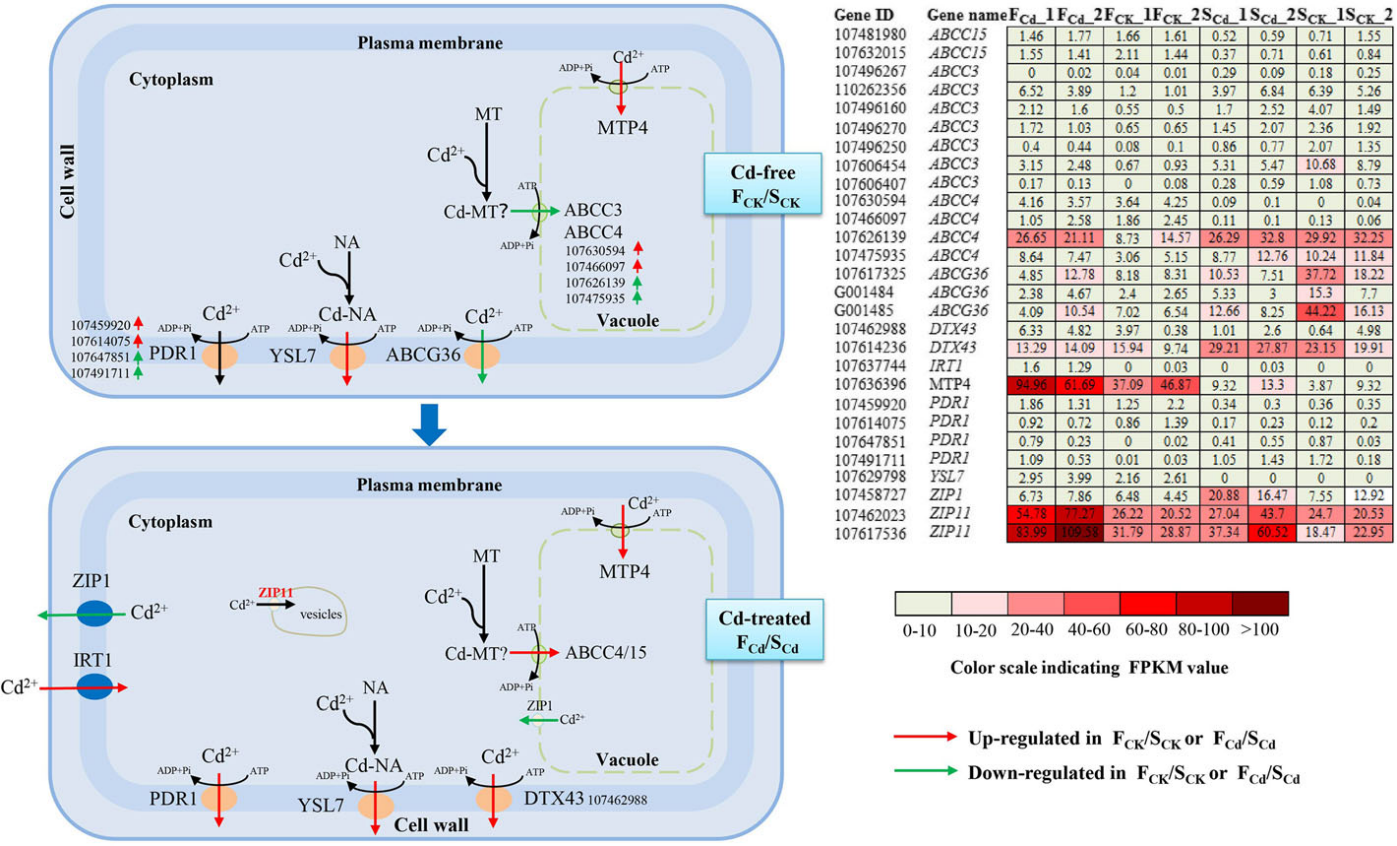
A schematic representation of transport genes involved in differential Cd translocation and accumulation in Silihong and Fenghua 1 plants. Red font indicates the gene up-regulated by FPKM analysis. The grid representing the FPKM value of each gene in eight samples, respectively. The localization of these genes was based on the data of Arabidopsis, rice and other species (see Table 1)

We found that six DEGs encoding metal transporters related to Cd influx, including *IRT1*, *ZIP4* and *Nramp5/6* (Table 1). *AtIRT1* [31], *NcZNT1*/*AtZIP4* [20], *SaNramp6* [32] and *OsNramp5* [33] have been shown to mediate cellular metals (Zn, Fe, Mn and Cd) uptake across the plasma membrane. IRT1, a Fe-regulated transporter, is expressed in the root epidermis [34]. Overexpressing *AtIRT1* in *Arabidopsis* leds to increased accumulation of Cd and Zn in the transgenic lines under Fe starvation [31], which indicates that *AtIRT1* can affect the Cd uptake capacity. Therefore, under Cd exposure, the higher expression of *IRT1* in the roots of Fenghua 1 compared with Silihong may be involved in the difference of root Cd absorption between Fenghua 1 and Silihong (Figs. 5 and 6). Overexpression of *AtZIP4* and *SaNramp6* in *Arabidopsis* increased accumulation of Cd in transgenic plants [20, 32]. *AtNramp6* is also thought to be a major role in the intracellular distribution of Cd [35]. Our results showed that ZIP4, Nramp5 and IRT1 were up-regulated by Cd in Fenghua 1, while in Silihong, they were unchanged (Fig. 5; Table 1). *Nramp5* (107606033, 107470537 and 107460699) shows a high degree of homology to the *AtNramp6* from Arabidopsis. However, Nramp6 (107618045) was upregualted in Silihong, while in Fenghua 1, it was unchanged (Fig. 5; Table 1). ZIP4 and Nramp5/6 were not DEGs as judged by our criteria between the two cultivars. These results suggested that ZIP4 and Nramp5/6 may be related to Cd-response in roots of two peanut cultivars. However, the functions of these genes in peanuts need further investigated.

**Table 1:** Critical DEGs involved in Cd transport in roots of two peanut cultivars

Gene ID	Log_2_ fold-change				Description	Gene name	Functional analysis^b^	Reference
	F_Cd_/F_CK_	S_Cd_/S_CK_	F_Cd_/S_Cd_	F_CK_/S_CK_				
Plasma membrane-localized transporters								
107606033	1.80^a^	1.13	−0.03	− 0.78	metal transporter Nramp5	Nramp5	Transport Cd into cytoplasm	[19, 33]
107470537	2.01^a^	1.15	0.20	− 1.11	metal transporter Nramp5-like	Nramp5	Transport Cd into cytoplasm	[19, 33]
107460699	1.63^a^	0.91	− 0.02	− 0.80	metal transporter Nramp5-like	Nramp5	Transport Cd into cytoplasm	[19, 33]
107618045	0.79	1.25^a^	− 0.65	− 0.25	metal transporter Nramp6	Nramp6	Transport Cd into cytoplasm	[32]
107461206	1.17^a^	0.57	0.03	− 0.61	zinc transporter 4, chloroplastic	ZIP4	Transport Cd into cytoplasm	[20]
G003600	1.05^a^	0.60	− 0.13	−0.62	zinc transporter 4, chloroplastic	ZIP4	Transport Cd into cytoplasm	[20]
107637744	3.10^a^	0.12	4.03^a^	0.20	Fe (2+) transport protein 1	IRT1	Transport Cd into cytoplasm	[31, 34]
107614121	1.09^a^	1.13	−0.01	0.04	zinc transporter 1	ZIP1	Export Cd out of cell	[46]
107458727	0.34	0.61	− 1.28^a^	−1.05	zinc transporter 1	ZIP1	Export Cd out of cell	[46]
107482454	1.42^a^	0.89	−0.42	−0.97	zinc transporter 1; zinc transporter 5	ZIP1/ZIP5	Export Cd out of cell	[46]
107638316	1.37^a^	0.64	−0.15	− 0.96	zinc transporter 5	ZIP5	Export Cd out of cell	[46]
107460374	1.33^a^	0.70	0.76	0.08	metal-nicotianamine transporter YSL3	YSL3	Export Cd out of cell	[43]
107605860	1.51^a^	0.81	0.93	0.19	metal-nicotianamine transporter YSL3	YSL3	Export Cd out of cell	[43]
107460375	1.01^a^	0.68	0.33	−0.04	metal-nicotianamine transporter YSL3	YSL3	Export Cd out of cell cytoplasm	[43]
107629798	0.44	N/A	6.18^a^	6.36^a^	metal-nicotianamine transporter YSL7 isoform X1	YSL7	Export Cd out of cell	[40]
G001484	0.36	− 1.57	−0.18	− 2.31^a^	ABC transporter G family member 36	ABCG36	Export Cd out of cell	[41]
107617325	0.03	−1.75	0.00	−1.93^a^	ABC transporter G family member 36-like	ABCG36	Export Cd out of cell	[41]
G001485	0.04	−1.61	−0.44	− 2.28^a^	ABC transporter G family member 36-like	ABCG36	Export Cd out of cell	[41]
107459920	−0.15	− 0.38	2.20^a^	1.94^a^	pleiotropic drug resistance protein 1	PDR1	Export Cd out of cell	[41, 42]
107647851	2.76^a^	−0.20	0.11	− 3.44^a^	pleiotropic drug resistance protein 1	PDR1	Export Cd out of cell	[41, 42]
107491711	3.49^a^	−0.04	−0.52	−4.52^a^	pleiotropic drug resistance protein 1-like	PDR1	Export Cd out of cell	[41, 42]
107614075	−0.43	0.06	1.90^a^	2.45^a^	pleiotropic drug resistance protein 1-like	PDR1	Export Cd out of cell	[41, 42]
107472005	1.18^a^	0.68	−0.24	−0.77	metal tolerance protein 9	MTP9	Export Cd out of cell	[38]
107622321	1.24^a^	0.86	−0.38	− 0.81	metal tolerance protein 9	MTP9	Export Cd out of cell	[38]
107614236	0.06	0.11	−1.00^a^	− 0.97	protein DETOXIFICATION 43	DTX43	Export Cd out of cell	[44, 45]
107462988	0.91	− 0.55	1.49^a^	− 0.38	protein DETOXIFICATION 43-like X1	DTX43	Export Cd out of cell	[44, 45]
Tonoplast-localized transporters								
107481980	−0.06	−1.12	1.50^a^	0.36	ABC transporter C family member 15	ABCC15	Sequestrate Cd into vacuole	[21]
107632015	−0.27	−0.61	1.38^a^	1.03	ABC transporter C family member 15	ABCC15	Sequestrate Cd into vacuole	[21]
107496267	−0.18	−0.34	−2.11	− 2.83^a^	ABC transporter C family member 3	ABCC3	Sequestrate Cd into vacuole	[21]
107496250	1.63	−1.25	− 0.86	−4.15^a^	ABC transporter C family member 3-like	ABCC3	Sequestrate Cd into vacuole	[21]
107496160	1.65^a^	− 0.62	− 0.15	−2.52^a^	ABC transporter C family member 3-like	ABCC3	Sequestrate Cd into vacuole	[21]
110262356	2.02^a^	− 0.29	−0.01	− 2.56^a^	ABC transporter C family member 3-like	ABCC3	Sequestrate Cd into vacuole	[21]
107606454	1.58^a^	− 1.08	− 0.87	−3.66^a^	ABC transporter C family member 3-like	ABCC3	Sequestrate Cd into vacuole	[21]
107496270	0.95	− 0.52	− 0.29	− 1.92^a^	ABC transporter C family member 3-like	ABCC3	Sequestrate Cd into vacuole	[21]
107606407	0.34	− 1.08	−0.99	−3.49^a^	ABC transporter C family member 3-like	ABCC3	Sequestrate Cd into vacuole	[21]
107630594	−0.05	0.74	4.48^a^	5.84^a^	ABC transporter C family member 4 X3	ABCC4	Sequestrate Cd into vacuole	[36]
107466097	−0.26	−0.10	3.46^a^	3.94^a^	ABC transporter C family member 4 X3	ABCC4	Sequestrate Cd into vacuole	[36]
107475935	0.83	−0.28	−0.37	−1.55^a^	ABC transporter C family member 4-like	ABCC4	Sequestrate Cd into vacuole	[36]
107626139	0.88	−0.33^a^	− 0.26	− 1.55^a^	ABC transporter C family member 4-like	ABCC4	Sequestrate Cd into vacuole	[36]
107636396	0.78	0.54	2.70^a^	2.42^a^	metal tolerance protein 4	MTP4	Sequestrate Cd into vacuole	[37]
Endomembrane-localized transporters								
107461527	1.33^a^	1.14	−0.03	− 0.25	zinc transporter 1	ZIP1	Sequestrate Cd into vesicles	[28]
107462023	1.37^a^	0.33	0.89	− 0.21	zinc transporter 11	ZIP11	Sequestrate Cd into vesicles	[28]
107617536	1.55^a^	0.93	0.97	0.33	zinc transporter 11	ZIP11	Sequestrate Cd into vesicles	[28]
107618045	0.79	1.25^a^	−0.65	− 0.25	metal transporter Nramp6	Nramp6	Sequestrate Cd into vesicles	[35]

^a^The gene differentially expressed between the two groups. ^b^Function analysis of DEGs was based on references listed in the same row

Furthermore, several DEGs encoding metal transporter proteins related to sequestration of Cd into vacuolar (ABCC3/4 and MTP4) and the endomembrane systems (ZIP11) are also observed (Table 1). The tonoplast-localized AtABCC3/AtMRP3 has been reported to regulated Cd detoxification via sequestration of PC-Cd complexes into vacuolar [21]. AtMRP4/AtABCC4 mediate the transport of folate into vacuolar [36]. Here, although most genes encoding ABCC3 were up-regulated by Cd in Fenghua 1 (Fig. 5), they were unchanged between Fenghua 1 and Silihong under Cd exposure (Table 1). This result suggests that ABCC3 may not be involved in the difference of shoot Cd accumulation between the two cultivars. Two *ABCC15* (107481980, 107632015) genes were close homologue of *AtMRP3* in *Arabidopsis* (Additional file 12: Table S10). Importantly, ABCC4 (107466097, 107630594), ABCC15 (107481980, 107632015) and *MTP4* showed higher expression in Fenghua 1 roots than in Silihong roots under Cd exposure (Fig. 6; Table 1). *CsMTP4* was shown to function in Zn and Cd transport and tolerance by sequestering Cd into the vacuolar compartments in cucumber [37]. Xu et al. [28] reported that ZIP11 is located in the endomembrane, which could compartmentalize Zn/Cd into vesicles and prevent metal translocation from root to shoot. Here, two ZIP11 (107462023, 107617536) genes were up-regulated by Cd in Fenghua 1, while in Silihong, they were unchanged (Fig. 5; Table 1). FPKM value analysis indicated that ZIP11 (107462023) showed higher expression in Fenghua 1 roots than in Silihong roots (Fig. 6). These results suggest that Cd-inducible *ABCC4*, *ABCC15, MTP4* and ZIP11 may enhance root vacuoles or vesicles sequestration of Cd and consequently, reducing root-to-shoot Cd translocation in Fenghua 1. Whether these genes associate with sequestration of Cd in peanut plants or not require further studies.

We also identified some DEGs encoding plasma membrane-localized transporter proteins related to Cd efflux, including ABCG36/PDR8, PDR1, YSL3/7, MTP9, DTX43 and ZIP1/5 (Table 1). These genes have been reported to regulate in Cd accumulation or detoxification in other plants [38–41]. Migocka et al. [38] reported that CsMTP9 in cucumber was a plasma membrane H^+^-coupled antiporter involved in the Mn^2+^ and Cd^2+^ efflux from root cells. Here, MTP9 was up-regulated by Cd in Fenghua 1, but in Silihong, it was unchanged, and it showed no significant differential expression between the two cultivars under control and Cd-treated (Fig. 5; Table 1). Peanut PDR 1 and DTX43 were homology with AtPDR12 and AtFRD3 in *Arabidopsis*, respectively (Additional file 12: Table S10). Two PDR 1 (107647851, 107491711) were upregulated in Fenghua 1, but in Silihong, it was unchanged. Besides, PDR1, DTX43 (107462988) and YSL7 transporters showed higher expression in Fenghua 1 than in Silihong under Cd exposure (Fig. 6; Table 1). This observation is also consistent with previous reports that the expression of *YSL7* and *MATE efflux family protein* indicated higher expression in QLQ (low-shoot-Cd) than in T308 (high-shoot-Cd) [27]. In Arabidopsis, two ABC genes, *AtABCG36/PDR8* and *AtABCG40/PDR12*, are induced by Cd as an efflux pump of Cd^2+^ conferring detoxification of Cd [40–42]. In contrast, ABCG36/PDR8 showed higher expression in Silihong than in Fenghua 1 under control condition, while it was unchanged under Cd-treated (Fig. 6; Table 1). Further, plasma membranes-localized YSL transporter family can involve in transporting of NA-metal complexes, and *BjYSL7* [39] and *SnYSL3* [43] have been reported to participate in the efflux of NA-Cd complexes. YSL3 was upregulated by Cd in Fenghua 1 (Fig. 5; Table 1), but no difference was observed between two cultivars. FRD3, a MATE efflux family protein, is thought to be involved in Cd tolerance via the removing Cd from the cytoplasm [44, 45]. These results indicated that PDR1, DTX43 and YSL7 may be the critical factors in determining the contrasting shoot-Cd accumulation in two cultivars. In addition to genes mentioned above, four genes belonging to ZIP family, ZIP1 (107461527, 107614121 and 107482454) and ZIP 5 (107638316) were upregulated by Cd in Fenghua 1, but in Silihong, it was unchanged (Fig. 5; Table 1). ZIP1 (107614121) and ZIP1 (107482454) of peanut exhibit high sequence similarity to *Arabidopsis* AtZIP2 and peanut ZIP5, respectively (Additional file 12: Table S10). Milner et al. [46] reported that AtZIP1 was localized at the vacuolar membrane, while AtZIP2 was localized to plasma membrane of root stele cells, and both transporters play a role in Mn and Zn translocation from roots to shoots. ZIP1 (107458727) shows lower expression in Fenghua 1 than in Silihong under Cd exposure (Fig. 6; Table 1). Therefore, the lower expression of ZIP1 in the roots of Fenghua 1 may reduce Cd translocation from roots to shoots, and the exact role of this transporter needs further investigation.

### Retention of Cd in the root may contribute to the contrasting shoot-Cd accumulation in Fenghua 1 and Silihong

Cell wall is the first barrier to Cd uptake into the cytosol [47]. It is also the major site for Cd^2+^ binding and retention in plant roots [48]. The Cd binding capacity of root cell walls could influence the cultivar difference in Cd translocation to the shoot [27]. In this study, a total of 260 DEGs (F_Cd_/F_CK_: 27; S_Cd_/S_CK_: 40; F_Cd_/S_Cd_: 93; F_CK_/S_CK_: 191) were identified to show high similarity with cell wall-related proteins (Additional file 14: Table S12).

Twenty two genes encoding enzymes involved in the biosynthesis of cellulose, hemicellulose, lignin and suberin were up-regulated by Cd in Fenghua 1, including 13 UDP-glycosyltransferase family genes (e.g., UGT71K1, UGT72D2 and UGT83A1), four cytochrome P450 family genes (CYP450s), three 4-coumarate: CoA ligase-like proteins (4CL9), cellulose synthase-like protein G2 (CSLG2) and caffeoyl-CoA 3-O-methyltransferase (CCoAOMT) (Additional file 14: Table S12). In contrast, only one gene encoding suberin synthesis enzymes (CYP450 704C1, 107475575) was up-regulated by Cd in Silihong, while the remained 21 DEGs (95.24%) encoding cellulose, lignin and suberin biosynthesis enzymes were down-regulated (Additional file 14: Table S12). These results suggested that the cell wall biosynthesis was more active in Fenghua 1 than in Silihong, and this may lead to different binding capacity of Cd to cell walls.

It has been reported that pectin and hemicellulose components of the cell wall can bind Cd^2+^ by the negatively charged carboxyl and hydroxyl groups [47]. Thus, increased pectin and hemicellulose content might prevent the translocation of Cd from the roots to shoots [48]. PME catalyzes pectin demethylation, which enhances the capacity of pectin to bind metal ions [49]. Overexpression *OsPME14* in transgenic rice increased Al retention in the root tip cell wall [50]. Polygalacturonase inhibitor proteins (PGIPs) can inhibit polygalacturonase (PG) activity and pectin-degrading. Moreover, xyloglucan hemicellulose component has been reported to be a much more effective binding of Al than pectin in *Arabidopsis* [51]. Here, most of DEGs (59/93, 63.44%) were expressed higher in Fenghua 1 than that in Silihong under Cd-treated conditions, including 24 DEGs involved in pectin catabolic process, eight DEGs involved in suberin synthesis and cellulose catabolic process, respectively, seven DEGs involved in hemicellulose catabolic process, five DEGs involved in lignin synthesis, one DEG involved in the formation of Casparian strip and the other three involved in cell wall modification (Additional file 14: Table S12). Among of them, the higher expression of pectinesterase (PEs, 107475981, 107630347), PGIPs (i.e., 107481713, 107481714 and 107631575), xyloglucan glycosyltransferase 12 (XYT12) and glycosyltransferases (GTs, 107638833, 107459345) in Fenghua 1 might increase the retention of Cd in root cell wall and consequently, reducing Cd influx into the cytosol.

Furthermore, the intensive wall thickenings were observed in the inner tangential walls of endodermal cells after exposure to Cd [52], which could cause by the formation of Casparian strips and suberin lamellae, increase of suberin and lignin contents. These changes can serve as a barrier limiting apoplasmic flow of Cd to xylem and its translocation to shoots [53, 54]. In this study, the higher expression of casparian strip membrane protein 5 (CASP5, 107634327), CYP450s (i.e., 107475575, 107478900, and 107482119), 4-coumarate--CoA ligase 2 (4CL2), laccases (LACs, 107639475, 107496989 and 107625654) and cinnamate 4-monooxygenase (C4H) in Fenghua 1 may contribute to cell wall modification in the roots and consequently leading to lower root-to-shoot Cd translocation.

The retention of Cd in the roots can be mediated by metal-chelating molecules such as MTs, GSHs, PCs and NAs [25, 55]. In this study, NAS (107612042) was upregulated by Cd in Fenghua 1, while in Silihong, it was unchanged. Moreover, NAS (107612042) and MT2 (107639467) were expressed higher in Fenghua 1 than in Silihong under Cd exposure (Additional file 14: Table S12), suggesting that these two genes may be involved in the difference of root Cd sequestration between Fenghua 1 and Silihong. However, no significant difference in GSH and PC biosynthesis-related genes expression were observed in each comparison, suggesting that GSHs and PCs may not be the key factors regulating the cultivar difference in Cd retention in peanut roots.

## Conclusions

In this study, 183 DEGs encoding ion transport related proteins (i.e., ZIPs, ABCs, Nramps, YSLs and MTPs) were identified. Importantly, one Cd influx gene (*IRT1*), four Cd efflux genes (*PDR12*, *YSL7*, ZIP1 and *FRD3*) and four tonoplast- and endomembrane-localized transport genes (*ZIP11*, *MTP4*, *ABCC4* and *ABCC15*), showed higher expression in Fenghua 1 than in Silihong, might be responsible for the cultivar differences in shoot Cd accumulation in peanut. Furthermore, 260 DEGs encoding cell wall-related proteins were also identified. Cell wall biosynthesis may be more activated in Fenghua 1. The higher expression of *PEs*, *PGIPs*, *GTs*, *XYT12 CYP450s*, *LACs*, *4CL2*, *C4H* and *CASP5* genes in Fenghua1 might contribute to reducing movement of Cd to xylem and its translocation to shoots, thus these genes might be involved in cultivar difference in shoot Cd accumulation of peanut. These results could provide a new insight into the molecular mechanisms about cultivar difference in Cd accumulation, which would be essential for breeding low-Cd cultivars of peanuts.

## Methods

### Plant materials and experiment design

According to our previous study [8], two peanut cultivars differing in seed Cd concentrations, Fenghua 1 (F, low-Cd cultivar) and Silihong (S, high-Cd cultivar) were selected for this experiment. Seeds of the two peanut cultivars were obtained commercially from the Peanut Institute of Shandong Province, Qingdao, China. Seeds were surface sterilized in 5% sodium hypochlorite for 1 min, then soaked in distilled water for 24 h, and sown into pots with washed sand. One week after sowing, the seedlings with uniform size were transferred to polyethylene pot (six seedlings per pot) irrigated with 3.5 L of nutrient solution (pH 5.8) for two weeks. Then, the seedlings for each cultivar were treated with 0 (Cd-free) and 2 μM CdCl_2_ (Cd-treated), respectively. The experiment was arranged in a randomized complete design with three replications (pots). Peanut plants were cultured according to the conditions used by Su et al. [17]. After one week of treatment, root samples for RNA-seq and RT-qPCR analysis were collected separately from each Cd-free and Cd-treated seedling. A total of five independent biological replicates were sampled from a pool of six seedlings for RNA-Seq (two biological replicates) and RT-qPCR validation (three biological replicates). All samples were immediately frozen in liquid nitrogen and stored at − 80 °C. The Cd concentration of roots and shoots was determined by atomic absorption spectrometry as previously described [17].

### cDNA library construction and RNA sequencing

Total RNA was isolated from Cd-free and Cd-treated peanut roots of both cultivars using Trizol® Reagent (Invitrogen, Carlsbad, CA, USA) following the manufacturer’s instructions. Then the RNA integrity and purity were assessed using Agilent 2100 Bioanalyzer (Agilent, USA) and NanoDrop™ spectrophotometer (Thermo Fisher Scientific, USA), respectively. Eight cDNA libraries named F_CK__1, F_CK__2, F_Cd__1, F_Cd__2, S_CK__1, S_CK__2, S_Cd__1 and S_Cd__2 were constructed with the BGISEQ-500 platform combining the DNA nanoball-based nanoarrays and stepwise sequencing using combinational probe-anchor synthesis sequencing method (BGI, Shenzhen, China). Briefly, Poly (A) mRNAs were captured from total RNA with poly-T oligo-attached magnetic beads (Thermo Fisher Scientific, USA), and then were fragmented into small pieces using divalent cations and heat treatments. Next, the first-strand cDNA was synthesized using reverse transcriptase and random primers with small fragments of mRNA as templates; and the second-strand cDNA was synthesized via DNA polymerase I and RNase H. Thereafter, the double-stranded cDNA ends were further repaired by a single nucleotide ‘A’ base addition, and subsequent ligation of the adapter. After PCR amplification, the PCR yields were quantified by Qubit (Invitrogen) and pooled samples together to make a single strand DNA circle (ssDNA circle), which gave the final cDNA library.

DNA nanoballs (DNBs) were generated with the ssDNA circle by rolling circle replication (RCR) to enlarge the fluorescent signals at the sequencing process. The DNBs were loaded into the patterned nanoarrays and pair-end reads of 100 bp were read through on the BGISEQ-500 platform for the following data analysis study.

### Bioinformatics analysis

#### Data filtering and mapping

Using SOAPnuke software (BGI, Shenzhen, China), the raw reads were initially processed by removing reads containing adaptors, those with more than 5% unknown N bases and low-quality reads, and the clean reads in each library were obtained as the basis of further data analysis. Then, the clean reads were aligned to the reference genome sequences of peanut (*A. duranensis*-AA and *A. ipaensis*-BB) (Aradu1.1, https://www.ncbi.nlm.nih.gov/assembly/GCF_000817695.2; Araip1.0, https://www.ncbi.nlm.nih.gov/assembly/GCA_000816755.1) [29] using HISAT2 (v2.2.4, http://www.ccb.jhu.edu/software/hisat) with default parameters. The mapping reads for each library were reconstructed using StringTie software (v1.0.4, http://ccb.jhu.edu/software/stringtie); and then Cuffmerge was used to integrate all the reconstructed transcripts from the eight libraries. Novel transcripts were obtained by comparing reconstructed transcripts to reference annotation using Cuffcompare (Cufflinks v2.2.1, http://cole-trapnell-lab.github.io/cufflinks); and then, the coding potential of novel transcripts were predicted using CPC (v0.9-r2, http://cpc.cbi.pku.edu.cn); and a complete reference was generated via merging the novel coding transcripts with the reference transcripts. Finally, the clean reads were aligned to the complete reference using Bowtie2 program (v2.2.5, http://bowtie-bio.sourceforge.net/bowtie2/index.shtml).

#### Gene expression analysis

Expected count tables for each gene were obtained by RSEM (v1.2.12, http://deweylab.biostat.wisc.edu/RSEM) package. The expression levels for each of the gene in each library were normalized to FPKM (fragments per kilobase of transcript per million mapped reads). Based on the negative binomial distribution, read counts were used to determine the differential expression analysis among four groups (F_CK_, F_Cd_, S_CK_ and S_Cd_, two biological replicates per group) by the DEseq 2 package as described by Love et al. [56]. The |log_2_ fold-change| ≥ 1 and *P*_adj_-value ≤ 0.05 were used as thresholds to determine the significance of the difference in gene expression.

#### Gene ontology (GO) and pathway analysis of DEGs

With GO and pathway annotation result, the GO and KEGG functional classification for the DEGs were performed according to official classification, respectively. Then, GO and KEGG pathway enrichment analysis was implemented using phyper in R package with a corrected *p*-value ≤ 0.01 to identify significantly enriched GO terms and biological pathways.

### RT-qPCR validation

Total RNA was isolated from three biological replicates of root samples of both peanut cultivars exposed to 0 and 2 μM CdCl_2_ treatments, respectively. Then the first strand cDNA was synthesized by Prime Script® RT reagent Kit (Takara, Dalian, China). The primers of 16 DEGs were designed using Beacon Designer 7.0 software (Premier Biosoft International, USA), and *Actin* gene was used as the internal control (Additional file 15: Table S13). The primer specificity was evaluated by BLASTing primer sequences against the NCBI database. RT-qPCR was performed using a SYBR Premix EX Taq Kit (Takara) in a 20 μl reaction mixture on an ABI7300 (Applied Biosystems, Foster City, CA, USA) following the method described by Yu et al. [26]. All reactions were performed in three technical replicates. The equation ratio = 2^−ΔΔ*C*τ^ was employed to calculate the relative expression level of the selected genes. Data statistical analysis with IBM SPSS Statistics Version 25 software (Armonk, NY, USA) was conducted using Duncan’s multiple range test at the *P* < 0.05 level of significance.

## Additional files

Additional file 1: **Table S1.** Overview of raw and clean reads in Cd-free (CK) and Cd-treated (Cd) for two peanut cultivars. (XLSX 10 kb)

Additional file 2: **Table S2.** Mapping results of clean reads against the peanut reference sequence. (XLSX 11 kb)

Additional file 3: **Table S3.** Summary of novel transcripts. (XLSX 9 kb)

Additional file 4: **Table S4.** Gene expression analysis for all sample libraries. (XLSX 5503 kb)

Additional file 5: **Figure S1.** The gene expression distribution (FPKM) in eight samples. X axis represents the sample name. Y axis represents the gene amount. The dark color means the moderate and high expression level which FPKM value ≥10, while the light color means the low expression level which FPKM value < 10. (DOCX 282 kb)

Additional file 6: **Table S5.** List of differentially expressed genes in four groups based on pairwise comparisons. (XLSX 27764 kb)

Additional file 7: **Figure S2.** Gene ontology classification of DEGs identified. The enriched biological process, cellular component and molecular function GO terms of Cd-responsive DEGs in two peanut cultivars (a) and DEGs between Fenghua and Silihong under CK and Cd-treated conditions (b). The x-axis represents the GO term; the y-axis denotes the number of genes. (DOCX 314 kb)

Additional file 8: **Table S6.** GO enrichment analysis of all DEGs. The GO terms with corrected *p*-value ≤0.01 were considered significantly enriched among the DEGs. (XLSX 15 kb)

Additional file 9: **Table S7.** Overview of all 133 KEGG pathways for DEGs in four comparisons. (XLSX 33 kb)

Additional file 10: **Table S8.** List of enriched pathways for DEGs in four comparisons. Pathways with a corrected *P*-value ≤ 0.01 were regard as significantly enriched pathways in DEGs. (XLSX 12 kb)

Additional file 11: **Table S9.** The analysis of DEGs in F_Cd_/S_Cd_ with an FPKM value ≥10 in at least one of four cDNA libraries and |log_2_ (F_Cd_/S_Cd_)| ≥ 3. (XLSX 41 kb)

Additional file 12: **Table S10.** The DEGs involved in transport activities in two peanut cultivars under Cd-free and Cd-treated. Items highlighted in red mean up-regulated, while in green mean down-regulated. (XLSX 59 kb)

Additional file 13: **Table S11.** The seven expression patterns of transport-related DEGs in two peanut cultivars under Cd-free and Cd-treated. Items highlighted in red mean up-regulated, while in green mean down-regulated. (XLSX 28 kb)

Additional file 14: **Table S12.** The DEGs involved in cell wall metabolism and Cd chelating in two peanut cultivars. Items highlighted in red mean up-regulated, while in green mean down-regulated. (XLSX 71 kb)

Additional file 15: **Table S13.** The primers used in RT-qPCR analysis. (XLSX 13 kb)

## Abbreviations

4CL: 4-coumarate: CoA ligase-like protein
ABC: ATP-binding cassette transporter protein
CASP: Casparian strip membrane protein
CCoAOMT: Caffeoyl-CoA 3-O-methyltransferase
Cd: Cadmium
CDF: Cation diffusion facilitator
CHX: Cation/H+ exchanger
COPT: Copper transporter
CSL: Cellulose synthase-like protein
DEG: Differentially expressed gene
DTX: protein DETOXIFICATION
GO: Gene ontology
GSH: Glutathione
GT: Glycosyltransferase
HMA: Cadmium/zinc/copper-transporting ATPase (heavy metal-associated domain)
KEGG: Kyoto encyclopedia of genes and genomes
LAC: Laccase
MT: Metallothionein
MTP: Metal tolerance protein
NA: Nicotianamine
Nramp: Natural resistance-associated macrophage protein
OPT: Oligopeptide transporter
PC: Phytochelatin
PDR: Pleiotropic drug resistance-type ABC transporter protein
PE: Pectinesterase
PGIP: Polygalacturonase inhibitor protein
RT-qPCR: Reverse-transcription quantitative PCR
XYT: Xyloglucan glycosyltransferase
YSL: Yellow stripe-like transporter
ZIP: Zinc-regulated transporter/Iron-regulated transporter-like Protein
