# Supplementary material for: Comparative transcriptome analysis revealed key factors for differential cadmium transport and retention in roots of two contrasting peanut cultivars
Source: BMC Genomics. 2018 Dec 17;19:938. doi: 10.1186/s12864-018-5304-7 (PMC6296094; doi:10.1186/s12864-018-5304-7)
Supplement: Supplementary file 5 — Figure S1. The gene expression distribution (FPKM) in eight samples. X axis represents the sample name. Y axis represents the gene amount. The dark color means the moderate and high expression level which FPKM value ≥10, while the light color means the low expression level which FPKM value < 10. (DOCX 282 kb) [file 12864_2018_5304_MOESM5_ESM.docx]

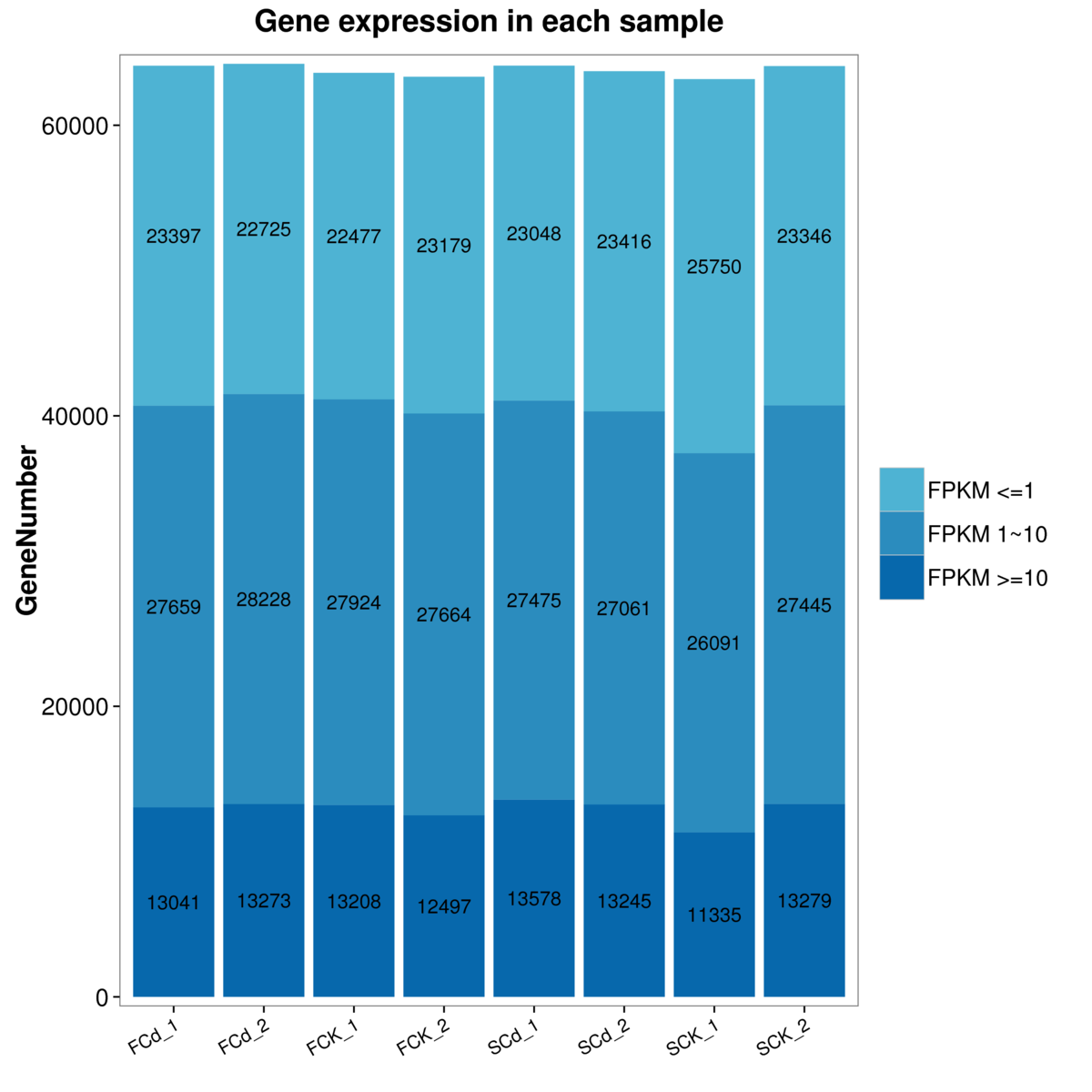


**Additional file 5: Figure S1.** **The gene expression distribution (FPKM) in eight samples.** X axis represents the sample name. Y axis represents the gene amount. The dark color means the moderate and high expression level which FPKM value ≥ 10, while the light color means the low expression level which FPKM value <10.
