# Supplementary material for: Comparative transcriptome analysis revealed key factors for differential cadmium transport and retention in roots of two contrasting peanut cultivars
Source: BMC Genomics. 2018 Dec 17;19:938. doi: 10.1186/s12864-018-5304-7 (PMC6296094; doi:10.1186/s12864-018-5304-7)
Supplement: Supplementary file 7 — Figure S2. Gene ontology classification of DEGs identified. The enriched biological process, cellular component and molecular function GO terms of Cd-responsive DEGs in two peanut cultivars (a) and DEGs between Fenghua and Silihong under CK and Cd-treated conditions (b). The x-axis represents the GO term; the y-axis denotes the number of genes. (DOCX 314 kb) [file 12864_2018_5304_MOESM7_ESM.docx]

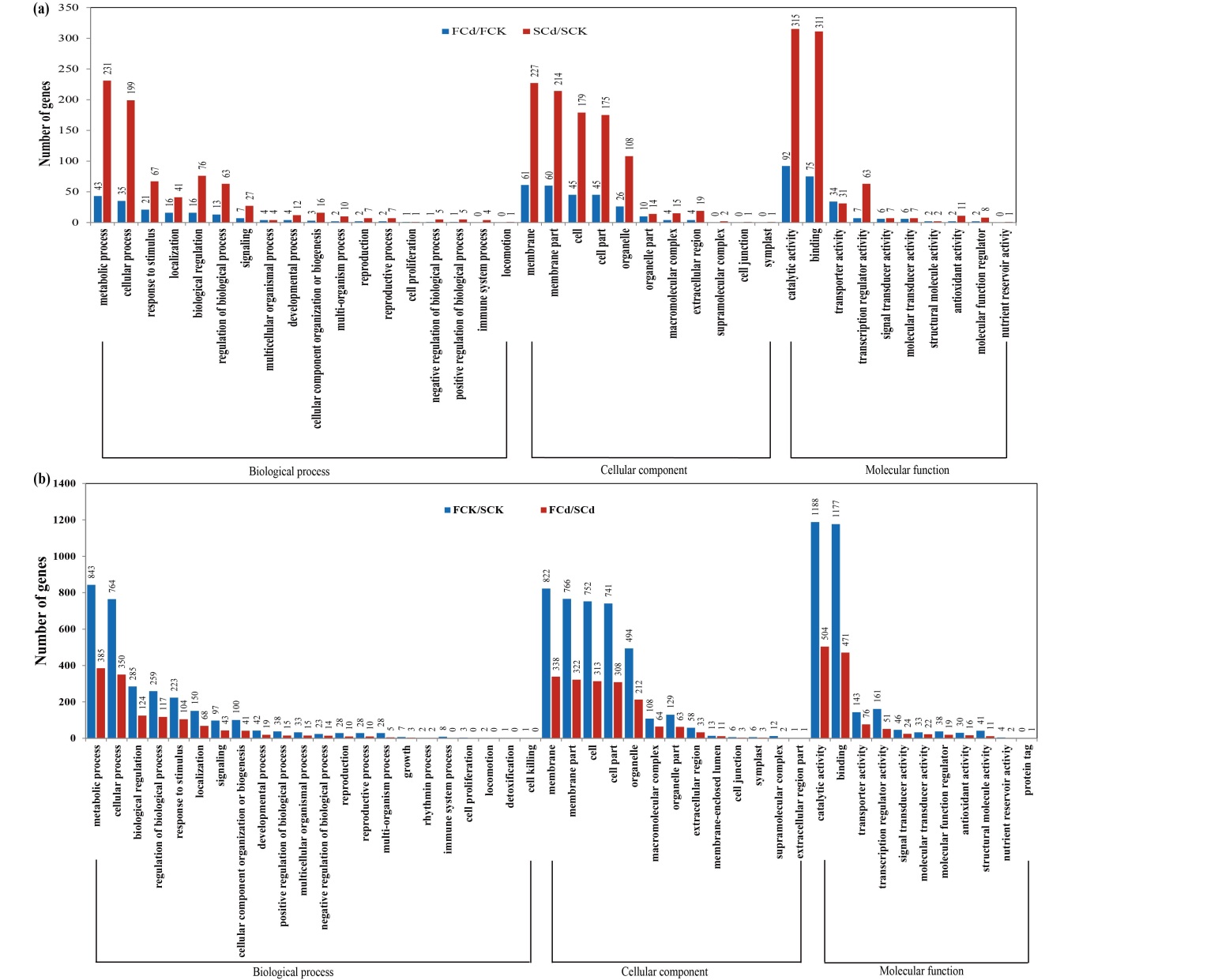


**Additional file 7: Figure S2.** **Gene ontology classification of DEGs identified.** The enriched biological process, cellular component and molecular function GO terms of Cd-responsive DEGs in two peanut cultivars (a) and DEGs between Fenghua and Silihong under CK and Cd-treated conditions (b). The x-axis represents the GO term; the y-axis denotes the number of genes.
